# Vital indicators in predicting burn mortality: A comparison of shock indices and burn shock indices

**DOI:** 10.55730/1300-0144.5758

**Published:** 2023-11-11

**Authors:** Mustafa İÇER, Ercan GÜNDÜZ, Cahfer GÜLOĞLU, Serkan ERBATUR, Dicle POLAT, Halime ÖZKAN, Tuğçe BAYRAK, Şilan GÖGER ÜLGÜT

**Affiliations:** 1 Department of Emergency Medicine, Dicle University Faculty of Medicine, Diyarbakır, Turkiye; 2 Department of Plastic and Reconstructive Surgery, Dicle University Faculty of Medicine, Diyarbakır, Turkiye; 3 Department of Emergency Medicine, Siverek Government Hospital, Şanlıurfa, Turkiye; 4 Department of Emergency Medicine, Silvan Government Hospital, Diyarbakır, Turkiye

**Keywords:** Burn, shock index, burn shock index, burn age shock index, BASI

## Abstract

**Background/aim:**

In many studies, shock indices have proven to be good tools for predicting mortality. In the present study, burn shock index (BSI), percentage of total body surface area burned (TBSA%) multiplied by shock index; burn modified shock index (BMSI), TBSA% multiplied by modified shock index; burn age shock index (BASI), TBSA% multiplied by age shock index; burn rivers shock index (BrSI), TBSA% multiplied by rivers shock index; burn rivers shock index multiplied by Glasgow Coma Scale score (BrSIG) were examined in burn patients. We defined these burn shock indices for the first time. This study aimed to evaluate the effectiveness of shock indices and burn shock indices in predicting mortality in burn patients.

**Materials and methods:**

This study examines retrospectively of burn patients admitted to the emergency department of Dicle University Hospital between January 2010 and December 2022. The patients’ vital signs were obtained at the time of presentation to the emergency department, and shock indices were calculated. The effectiveness of shock indices in predicting mortality was compared.

**Results:**

A total of 2445 patients were included in the study. Of the patients, 1793 were pediatric, and 652 were adults. BSI (AUC: 0.872, 95% confidence interval (CI): 0.812–0.931, p < 0.001) had the highest area under the curve (AUC) value in predicting mortality in children. The optimal cut-off value for BSI in children was 21.79 and its was sensitivity 83.05%, specificity 79.64%, positive predictive value (PPV) 12.19%, negative predictive value (NPV) 99.28%. In adults, BASI had the highest value of AUC (AUC: 0.936, 95% CI: 0.887–0.984, p < 0.001). The optimal cut-off value for BASI in adults was 62.5 and its sensitivity was 86.49%, specificity was 91.71%, PPV was 38.55%, and NPV was 99.12%.

**Conclusion:**

Shock indices are easy to calculate and effective in predicting mortality in burn patients admitted to the emergency department. Among the shock indices in the study, BSI was the best in predicting mortality in children, and BASI was the best in adults.

## 1. Introduction

Vital signs have an important role in evaluating patients admitted to the emergency department. Vital signs at admission significantly affect treatment, hospitalization, and intensive care unit admission. Heart rate (HR) and systolic blood pressure (SBP) are generally used to assess hemodynamic status.

Using HR and SBP, the shock index (SI) was first defined by Allgower et al. as the ratio of HR to SBP [1]. The normal range of SI is considered to be 0.5–0.7. Increased SI is associated with impaired hemodynamic status even when HR and SBP are within the normal range [2] SI, modified shock index (MSI), age shock index (ASI), rivers shock index (rSI), rSI multiplied by Glasgow Coma Scale score (GCS) (rSIG) have been studied in the field of triage, trauma, obstetrics, pediatrics, geriatrics, sepsis and pulmonary embolism. They determined that shock indices had a significant relationship with intensive care unit admission and mortality [2–5].

Burn injuries cause a systemic inflammatory response as a result of tissue damage, and many mediators (TNFα, interleukin-1, 2, 5, 8, and interferon γ, catecholamines, histamine, prostaglandins, thromboxane, nitric oxide) enter the circulation and cause hemodynamic deterioration and burn edema. In the first hours of burn injuries, burn shock results in a fluid shift from the intravascular space to the interstitium, including distributive, hypovolemic, and cardiogenic shock components [6].

The percentage of total body surface area burned (TBSA%) is considered a general prognostic marker in burn injuries. Mortality increases significantly when TBSA% is above 20% [7]. TBSA% is included in the scoring systems used to predict mortality in burn injuries and has an important prognostic value [8]. Fluid resuscitation in the first 24 h of burn injuries is one of the important treatment parameters affecting prognosis, and TBSA% is used to calculate the amount of fluid to be given [9]. Since TBSA% has such an important prognostic value in burn injuries, we considered it appropriate to use it in the burn shock indices we defined. We obtained burn shock indices by multiplying shock indices with TBSA%.

This study aimed to compare the effectiveness of SI, MSI, ASI, rSI, rSIG, burn shock index (BSI), burn modified shock index (BMSI), burn age shock index (BASI), burn rivers shock index (BrSI), burn rivers shock index times GCS (BrSIG) shock indices in predicting mortality in burn injuries.

## 2. Materials and methods

### 2.1. Study design and setting

This is a retrospective study of burn patients admitted to the emergency department of Dicle University Hospital. The emergency department of our hospital is a 3rd level emergency department; approximately 83,000 patients are admitted annually, and 26,500 of them are trauma patients. There is a 23-bed burn unit managed by plastic and reconstructive surgeons in our hospital.

### 2.2. Ethics statement

Approval was obtained from the Dicle University School of Medicine Ethics Committee for Non-interventional Clinical Research with session number 28.02.2023/62 before analyzing the data in the hospital registration system. Since the study was retrospective, patient consent was not obtained.

### 2.3. Emergency service management

All burn patients admitted to the emergency department were examined thoroughly, and vital signs were evaluated. TBSA% was calculated using the Lund Browder chart [10]. Fluid resuscitation was performed using the Parkland formula with a urine output of 0.5–1 ml/kg/hour [11]. All patients were resuscitated according to Advanced Trauma Life Support (ATLS) guidelines [12,13]. Patients were hospitalized in accordance with the hospitalization criteria of the American Burn Association [14].

### 2.4. Study population

This study analyzed 4855 consecutive burn patients admitted to the emergency department between January 2010 and December 2022. Burn patients who applied directly to our emergency department without applying to another emergency department and were hospitalized in the burn unit were included in the study.

**Figure 1:**
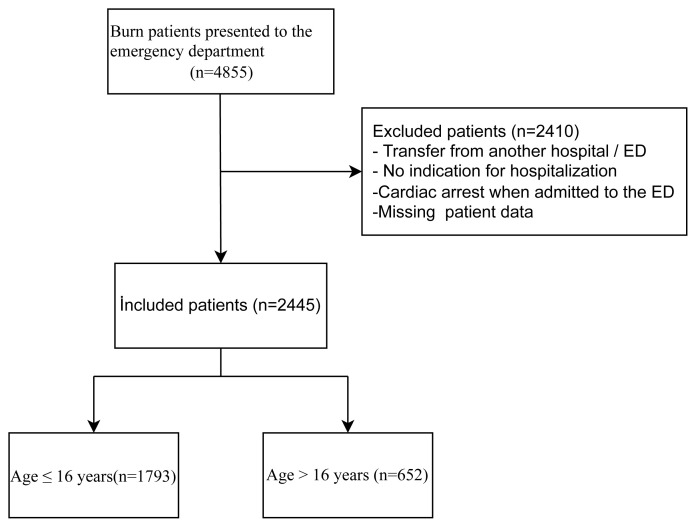
Flowchart of this study.

Exclusion criteria: Burn patients who were transferred to our hospital after being admitted to another emergency department or transferred from another hospital, who had a cardiac arrest when admitted to the emergency department, whose patient data were incomplete or inaccurate, and who were discharged from the emergency department without indication for hospitalization. Thus, 2410 patients were excluded, and 2445 patients were included in the study. The patients included in the study were divided into two groups as 16 years of age and younger (n =1793) and older than 16 years (n = 652) (Figure 1).

### 2.5. Data collection and variables

Patient information was obtained from the electronic hospital record system by reviewing patient files. The following parameters were recorded: age, sex, HR, SBP, diastolic blood pressure (DBP), mean arterial pressure (MAP), GCS, TBSA%, electrical burns, inhalation burns, intensive care unit stay, length of hospital stay, and survival. The patients’ vital signs at the time of presentation to the emergency department were evaluated. Then shock indices SI, MSI, ASI, rSI, rSIG, BSI, BMSI, BASI, BrSI, BrSIG were calculated.

### 2.6. Measurements

Shock indices were calculated using the following formulas.


$$\begin{array}{l} \text{SI}=\text{HR}/\text{SBP}\\ \text{MAP}=(\text{SBP}+2\times \text{DBP})/3\\ \text{MSI}=\text{HR}/\text{MAP}\\ \text{ASI}=\text{Age}\times \text{SI}\\ \text{rSI}=\text{SBP}/\text{HR}\\ \text{rSIG}=(\text{SBP}/\text{HR})\times \text{GCS}\\ \text{BSI}=\text{TBSA}\%\times \text{SI}\\ \text{BMSI}=\text{TBSA}\%\times \text{MSI}\\ \text{BASI}=\text{TBSA}\%\times \text{ASI}\\ \text{BrSI}=\text{TBSA}\%\times \text{rSI}\\ \text{BrSIG}=\text{TBSA}\%\times \text{rSIG} \end{array}$$

### 2.7. Statistical analyses

Numeric continuous variables with abnormal distribution were expressed as the median, interquartile range (IQR, q1-q3), and Mann-Whitney U-test were applied. Categorical variables were expressed as frequency and percentage, and the Chi-square test (χ2) was applied. The diagnostic decision-making properties of SI, MSI, ASI, rSI, rSIG, BSI, BMSI, BASI, BrSI, BrSIG shock indices in predicting mortality in burn patients were analyzed by receiver operating characteristic (ROC) curve analysis. The accuracy of the parameter in predicting mortality outcomes was defined as the area under the curve (AUC). The best cut-off point, sensitivity, specificity, positive predictive value (PPV), and negative predictive value (NPV) were determined based on the maximal Youden index. All tests were two-way, and p < 0.05 was considered statistically significant. The jamovi project (2022). jamovi (Version 2.3) [Computer Software]. Retrieved from https://www.jamovi.org Sydney, Australia was used for statistical analysis.

## 3. Results

### 3.1. Clinical characteristics and factors affecting mortality

Of the 2445 patients included in the study, 1793 (79.86%) were 16 years old or younger, and 652 (20.14%) were over 16 years old.

#### 3.1.1. Pediatric burn patients

Of 1793 burn patients, 1734 (96.71%) survived, and 59 (3.29%) died. The median age of pediatric burn patients was 2 (1–5) years in total, 2 (1–5) years in living patients, and 2 (1–3) years in dead patients (p = 0.646). Of the patients, 737 were female, and 1056 were male. The sex difference was not a factor affecting mortality (p = 0.201). Inhalation and electrical burns did not affect mortality (p = 0.169, p = 0.794, respectively). TBSA% was significantly higher in those who died. The median values of TBSA% were 10 (6–20) in total, 10 (6–17) in living patients, and 40 (25–60) in dead patients (p < 0.001), and it was a factor affecting mortality (p < 0.001). Of the 59 patients who died, 52 were intensive care unit patients. Intensive care hospitalization was a factor affecting mortality (p < 0.001). The length of hospital stay was shorter in those who died (p = 0.003) (Table 1).

DBP, SBP, MAP, GCS, rSI, and rSIG values were lower in the patients who died, and they were the factors affecting mortality (p < 0.001). Comparison of median (q1-q3) values in dead and living, respectively; DBP 54 (40–61.50) –60 (54–70), SBP 90 (80–106) –100 (91–113), MAP 66.33 (55.50–75.83)–74.33 (67.66–84), GCS 14 (10–15)–15 (15–15), rSI 0.73 (0.60–0.89)–0.88 (0.75–1.05), rSIG 9.10 (6.63–12.57)–13.12 (11.25–15.75). SI, MSI, BSI, BMSI, BASI, BrSI, and BrSIG values were higher in the patients who died, and they were the factors affecting mortality (p < 0.001). Comparison of median (q1-q3) values in dead and living, respectively; SI 1.36 (1.12–1.63)–1.13 (0.94–1.32), MSI 1.94 (1.44–2.37)–1.54 (1.29–1.80), BSI 48.93 (30.22–81.93)–11.83 (6.68–19.73), BMSI 75.50 (43.10–123.50)–16.14 (8.88–26.36), BASI 93.33 (39.95–229.15)–25.35 (11.50–63.63), BrSI 28.14 (18.94–40.38)–9.17 (5.15–15.82), BrSIG 300 (208–461.35)–136.46 (75.93–232.82). However, ASI did not affect mortality (p = 0.631) (Table 1).

#### 3.1.2. Adult burn patients

Of 652 burn patients, 615 (94.32%) survived, and 37 (5.68%) died. Age was higher in those who died. The median age of adult burn patients was 32 (23–43) years in total, 31 (23–43) years in survivors, and 46 (29–58) years in patients who died. Age was a factor affecting mortality (p < 0.001). There were 178 females and 474 males. Sex difference was not a factor affecting mortality (p = 0.595). Of inhalation burns, 167 (87.4%) survived, and 24 (12.6%) died. Of electrical burns, 244 (96.8%) survived, and 8 (3.2%) died. Inhalation and electrical burns were factors affecting mortality (p < 0.001, p = 0.044, respectively). TBSA% was significantly higher in those who died. The median values of TBSA% were 14.5 (6.25–25) in the total, 12 (6–20) in the living, and 55 (30–77.5) in the patients who died, and it was a factor affecting mortality (p < 0.001). Of the 37 patients who died, 33 were intensive care unit patients. Intensive care hospitalization was a factor affecting mortality (p < 0.001). The length of hospital stay was shorter in those who died and was a factor affecting mortality (p < 0.001) (Table 2).

DBP and MAP did not affect mortality (p = 0.215, p = 0.093, respectively). SBP, GCS, rSI, and rSIG were lower in those who died and were factors affecting mortality (p = 0.006, p < 0.001, p < 0.001, p < 0.001 respectively). Comparison of median (q1-q3) values in dead and living, respectively; SBP 112 (100–125)–120 (110–130), GCS 15 (12–15)–15 (15–15), rSI 1.08 (0.86–1.30)–1.35 (1.19–1.52), rSIG 15.68 (9.13–18.36)–20.06 (17.43–22.71). SI, MSI, ASI, BSI, BMSI, BASI, BrSI, and BrSIG were higher in the dead and were factors influencing mortality (p < 0.001). Comparison of median (q1-q3) values in dead and living, respectively; SI 0.92 (0.76–1.15)–0.73 (0.65–0.83), MSI 1.29 (1–1.55) –1 (0.88–1.13), ASI 45 (25.75–65.45)–23.67 (16.98–31.64), BSI 52.10 (26.55–79.17)–8.9 (4.66–16.69), BMSI 67.74 (37.47–109.09)–12.07 (6.27–23.15), BASI 92.73 (68.84–140.25)–35.10 (26.59–46.08), BrSI 53.96 (30.55–73.88)–16 (8.41–29.27), BrSIG 647.63 (325–990.47)–237.5 (122.12–420.30) (Table 2).

### 3.2. ROC analysis for predicting mortality with shock indexes

The diagnostic values of shock indices in predicting mortality based on the evaluation by ROC analysis are presented below.

#### 3.2.1. Pediatric burn patients

**Figure 2:**
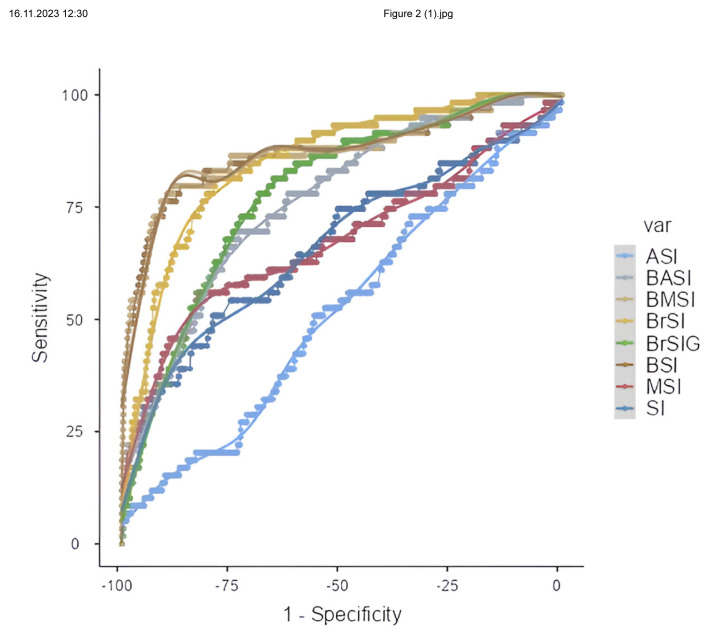
Receiver operating characteristic curve for shock indices and burn shock indices predicting mortality in pediatric burn patients.

As a result of the evaluation by ROC analysis, SI, MSI, rSI, rSIG, BSI, BMSI, BASI, BrSI, and BrSIG shock indices were found to have diagnostic value in predicting mortality. BSI and BMSI had the highest AUC values among the shock indices. In contrast, ASI had the lowest AUC value among the shock indices and had no diagnostic value in predicting mortality. AUC, 95% CI: lower bound-upper bound, p values of the shock indices; SI (AUC: 0.664, 95% CI: 0.582–0.746, p < 0.001), MSI (AUC: 0.675, 95% CI: 0. 589–0.761, p < 0.001), ASI (AUC: 0.518, 95% CI: 0.443–0.594, p = 0.631), rSI (AUC: 0.664, 95% CI: 0.582–0.746, p < 0.001), rSIG (AUC: 0.770, 95% CI: 0. 694–0.845, p < 0.001), BSI (AUC: 0.872, 95% CI: 0.812–0.931, p < 0.001), BMSI (AUC: 0.871, 95% CI: 0.812–0.931, p < 0.001), BASI (AUC: 0.764, 95% CI: 0.703–0.825, p < 0.001), BrSI (AUC: 0.851, 95% CI: 0.802–0.900, p < 0.001), BrSIG (AUC: 0.782, 95% CI: 0.726–0.837, p < 0.001) (Table 3). ROC curves of pediatric burn patients are given in Figure 2.

#### 3.2.2. Adult burn patients

**Figure 3:**
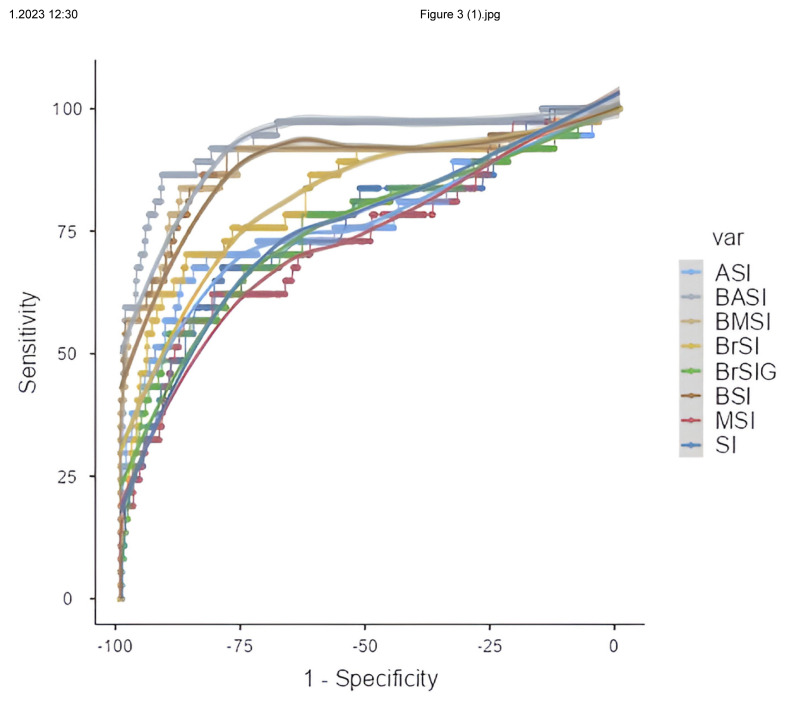
Receiver operating characteristic curve for shock indices and burn shock indices predicting mortality in adult burn patients.

As a result of the evaluation by ROC analysis, SI, MSI, ASI, rSI, rSIG, BSI, BMSI, BASI, BrSI, and BrSIG shock indices were found to have diagnostic values in predicting mortality. Among the shock indices, BASI had the highest AUC value. Then BSI and BMSI had the highest AUC values. AUC, 95% CI: lower bound-upper bound, p values of the shock indices; SI (AUC: 0.758, 95% CI: 0.668–0.847, p < 0.001), MSI (AUC: 0.735, 95% CI: 0. 642–0.829, p < 0.001), ASI (AUC: 0.772, 95% CI: 0.674–0.870, p < 0.001), rSI (AUC: 0.758, 95% CI: 0.668–0.847, p < 0.001), rSIG (AUC: 0.780, 95% CI: 0.693–0.867, p < 0.001), BSI (AUC: 0.888, 95% CI: 0.814–0.963, p < 0.001), BMSI (AUC: 0.885, 95% CI: 0.809–0.962, p < 0.001), BASI (AUC: 0.936, 95% CI: 0.887–0.984, p < 0.001), BrSI (AUC: 0.826, 95% CI: 0.745–0.907, p < 0.001), BrSIG (AUC: 0.755, 95% CI: 0.661–0.850, p < 0.001) (Table 4). ROC curves of adult burn patients are given in Figure 3.

## 4. Discussion

There are many studies proving the effectiveness of shock indices such as SI, MSI, ASI, rSI, and rSIG, including HR and SBP, on prognosis [2–5]. This study showed that the calculated values of known shock indices at the time of admission to the emergency department were effective in predicting mortality in burn patients. While burn patients show similarities and differences with trauma patients regarding etiology, physiopathology, emergency department practices, follow-up, and subsequent treatment stages, TBSA% is an important prognostic indicator specific to burn patients. In light of this basic information, this study proved the superiority of burn shock indices specific to burn patients, obtained by multiplying TBSA% with existing shock indices over existing shock indices in determining prognosis. Since shock indices in burn patients have not been studied before, the discussion was made by comparing with nonburn trauma studies due to the similarity between burn and trauma patients.

In pediatric trauma patients, SI and rSIG were found to be significant in predicting mortality [15,16]. In the study by Nazar et al. [17], the mortality rate was 14.89% when MSI was >1.3 in pediatric patients hospitalized in the intensive care unit, and it was significant in predicting mortality. Strutt et al. [15] classified pediatric trauma patients according to age groups as under one year, 1 to <2 years, 2 to <5 years, 5 to <12 years, and 12 to 14 years and took SI cut-off values as 2.7, 2.1, 1.9, 1.5, 1.1, respectively. In their study, they found the sensitivity of increased shock index in predicting mortality as 25.3% (19.4–32.2), specificity as 98.4% (98.2–98.5), PPV as 9.5% (7.2–12.5), and NPV as 99.5% (99.4–99.6). This study examined SI, MSI, rSI, rSIG, BSI, BMSI, BASI, BrSI, and BrSIG for all pediatric burn patients and was significant in predicting mortality. The AUC value of burn shock indices constructed using TBSA% increased significantly compared to SI, MSI, rSI, and rSIG. Among the shock indices, BSI was the best in predicting mortality in pediatric burn patients.

In a study evaluating patients admitted to the emergency department with multitrauma, SI was found to be an important predictor of mortality. The cut-off value of SI for mortality was 1.14, AUC: 0.738, 95% CI: 0.637–0.824, sensitivity 91.25%, specificity 53.85% [18]. In a study examining geriatric trauma patients admitted to the emergency department, the superiority of SI, MSI, and ASI in predicting mortality was proven. In the same study, the SI cut-off for 0.9 was sensitive to 49%, specificity of 95.4%; the MSI cut-off for 1.2 was sensitive to 49.5%, specificity of 95.2%; the ASI cut-off for 49 was sensitive to 73%, specificity of 74.9% in predicting hospital mortality for all patients [19]. SI, MSI, and ASI were effective in predicting mortality in emergency severity index level 3 adult patients admitted to the emergency department [20]. Bergen et al. [21] found that increased SI and MSI significantly reduced 30-day and one-year survival in adult patients admitted to the emergency department with out-of-hospital cardiac arrest. For SI cut-off 1, sensitivity was 27.3%, specificity 92%, PPV 77.9%, NPV 55%; for MSI cut-off 1.3, sensitivity 28.4%, specificity 91.2%, PPV 77.1%, NPV 59.4% for survival and discharge [21]. Kuo et al. [3] found that rSI < 1 in trauma patients admitted to the emergency department was significant for poor outcomes and trauma team activation in the emergency department. Kimura et al. [5] found the highest AUC value for hospital mortality in trauma patients under 55 years of age as 0.901, with 95% CI: 0.894–0.908 for rSIG. In a study conducted on trauma patients, SI, rSI, and rSIG were found to be significant in predicting mortality and AUC: 0.83, sensitive 94.5%, specificity of 61.5% for rSIG cut-off value of 14 and AUC: 0.63, sensitive 86.9%, specificity 38.1% for SI cut-off value 0.8 in patients without head trauma [22]. In this study, SI, MSI, ASI, rSI, rSIG, BSI, BMSI, BASI, BrSI, and BrSIG were analyzed for all adult burn patients and were significant in predicting mortality. The AUC value of burn shock indices constructed using TBSA% increased significantly. BASI was the best for predicting mortality for adult burn patients among the shock indices.

ASI is a shock index with proven efficacy in predicting prognosis in adults, especially in the geriatric population [19–23]. In this study, ASI was ineffective in predicting mortality in pediatric burn patients, whereas it was highly effective in burn patients over 16 years old. BASI constructed using TBSA% was the best in predicting mortality in adult burn patients.

## 5. Limitations

We recognized the following limitations in this study. First, the study is cross-sectional and retrospective; prospective validation studies are needed for validity. Nevertheless, we included all consecutive burn patients who met the inclusion criteria of the study. Second, the study is single-center and needs to be supported by international multicenter studies due to inter-regional differences worldwide. Third, since burn shock indices were used for the first time in burn patients, comparisons were made with nonburn patients. Therefore, burn shock indices need to be supported by other studies in the future.

## 6. Conclusions

Shock indices are scores with proven efficacy in predicting prognosis that can be easily calculated at the bedside without requiring additional devices. SI, MSI, ASI, rSI, and rSIG are significant in predicting mortality in burn patients. Burn shock indices BSI, BMSI, BASI, BrSI, BrSI, and BrSIG created using TBSA%, which have a strong effect on prognosis in burn patients, have a higher effect on predicting mortality. Among the shock indices, BSI was the best in predicting mortality in pediatric burn patients, and BASI was the best in adult burn patients.

## Figures and Tables

**Table 1:** Clinical characteristics, vital signs, shock indices and factors affecting mortality in pediatric burn patients.

Variables	Total (n = 1793)	Survival (n = 1734)	Mortality (n = 59)	p-value

Age, year (median[IQR])	2 (1–5)	2 (1–5)	2 (1–3)	0.646

Sex n(%)				
female	737 (100)	718 (97.4)	19(2.6)	0.201
male	1056 (100)	1016 (96.2)	40 (3.8)	

TBSA,(%),(median[IQR])	10 (6–20)	10 (6–17)	40 (25–60)	<0.001

Inhalation burn, n(%)	119 (100)	112 (94.1)	7 (5.9)	0.169

Electrical burn, n(%)	122 (100)	119 (97.5)	3 (2.5)	0.794

ICU admission, n(%)	888 (100)	836 (94.1)	52 (5.9)	<0.001

LOS,days (median[IQR])	10 (6–19)	10 (6–19)	7 (5–13)	0.003

Heart rate (beats/min), (median[IQR])	118 (103–128)	118 (103–128)	128 (102–140)	0.035

DBP (mmHg), (median[IQR])	60 (54–70)	60 (54–70)	54 (40–61.50)	<0.001

SBP (mmHg), (median[IQR])	100 (90–113)	100 (91–113)	90 (80–106)	<0.001

MAP (mmHg), (median[IQR])	74 (67.33–83.66)	74.33 (67.66–84)	66.33 (55.50–75.83)	<0.001

GCS, (median[IQR])	15 (15–15)	15 (15–15)	14 (10–15)	<0.001

SI, (median[IQR])	1.13 (0.95–1.33)	1.13 (0.94–1.32)	1.36 (1.12–1.63)	<0.001

MSI, (median[IQR])	1.55(1.29–1.81)	1.54 (1.29–1.80)	1.94 (1.44–2.37)	<0.001

ASI, (median[IQR])	2.46 (2.34–5.22)	2.45 (1.34–5.26)	2.82 (1.41–4.89)	0,631

rSI, (median[IQR])	0.87 (0.75–1.05)	0.88 (0.75–1.05)	0.73 (0.60–0.89)	<0.001

rSIG, (median[IQR])	13.03 (11.18–15.68)	13.12 (11.25–15.75)	9.10 (6.63–12.57)	<0.001

BSI, (median[IQR])	12.09 (6.76–20.63)	11.83 (6.68–19.73)	48.93 (30.22–81.93)	<0.001

BMSI, (median[IQR])	16.46 (9.14–27.53)	16.14 (8.88–26.36)	75.50 (43.10–123.50)	<0.001

BASI, (median[IQR])	26.11 (11.80–67.50)	25.35 (11.50–63.63)	93.33 (39.95–229.15)	<0.001

BrSI, (median[IQR])	9.50 (5.29–16.39)	9.17 (5.15–15.82)	28.14 (18.94–40.38)	<0.001

BrSIG, (median[IQR])	140.62 (78.75–241.52)	136.46 (75.93–232.82)	300 (208–461.35)	<0.001

**Abbreviations:** IQR, interquartile range ; TBSA%, percentage of total body surface area burned; ICU, intensive care unit stay; LOS, length of stay; DBP, diastolic blood pressure; SBP, systolic blood pressure; MAP, mean arterial pressure; GCS, glasgow coma scale; SI, shock index; MSI, modified shock index; ASI, age shock index; rSI, reverse shock index; rSIG, reverse shock index multiplied by glasgow coma scale; BSI, burn shock index; BMSI, burn modified shock index; BASI, burn age shock index; BrSI, burn reverse shock index; BrSIG, burn reverse shock index multiplied by glasgow coma scale

**Table 2:** Clinical features, vital signs, shock indexes, and factors affecting mortality in adult burn patients.

Variables	Total (n = 652)	Survival (n = 615)	Mortality (n = 37)	p-value

Age, year (median[IQR])	32 (23–43)	31 (23–43)	46(29–58)	<0.001

Sex n(%)				
female	178 (100)	166 (93.3)	12(6.7)	0.595
male	474 (100)	449 (94.7)	25 (5.3)	

TBSA(%),(median[IQR])	14.5 (6.25–25)	12 (6–20)	55 (30–77.5)	<0.001

Inhalation burn, n(%)	191 (100)	167(87.4)	24 (12.6)	<0.001

Electrical burn, n(%)	252 (100)	244 (96.8)	8 (3.2)	0.044

ICU admission, n(%)	404 (100)	371(91.8)	33 (8.2)	0.001

LOS,days (median[IQR])	13 (7–27)	13 (7–27)	6 (5–9)	<0.001

Heart rate (beats/min), (median[IQR])	88 (80–98)	88 (80–97)	110 (92–126)	<0.001

DBP (mmHg), (median[IQR])	70 (65–80)	70 (65–80)	70 (60–81)	0.215

SBP (mmHg), (median[IQR])	120 (110–130)	120 (110–130)	112 (100–125)	0.006

MAP (mmHg), (median[IQR])	88 (80.66–95.66)	88 (81–95.66)	83.33 (73.33–94)	0.093

GCS, (median[IQR])	15 (15–15)	15 (15–15)	15 (12–15)	<0.001

SI, (median[IQR])	0.74 (0.65–0.85)	0.73 (0.65–0.83)	0.92 (0.76–1.15)	<0.001

MSI, (median[IQR])	1.01 (0.89–1.15)	1 (0.88–1.13)	1.29 (1–1.55)	<0.001

ASI, (median[IQR])	24.12 (17.35–32.76)	23.67 (16.98–31.64)	45 (25.75–65.45)	<0.001

rSI, (median[IQR])	1.33 (1.17–1.51)	1.35 (1.19–1.52-)	1.08 (0.86–1.30)	<0.001

rSIG, (median[IQR])	19.79 (17.21–22.62)	20.06 (17.43–−22.71)	15.68 (9.13–18.36)	<0.001

BSI, (median[IQR])	9.46 (4.90–18.50)	8.9 (4.66–16.69)	52.10 (26.55–79.17)	<0.001

BMSI, (median[IQR])	12.91 (6.53–25.34)	12.07 (6.27–23.15)	67.74 (37.47–109.09)	<0.001

BASI, (median[IQR])	36.26 (26.86–48.59)	35.10 (26.59–46.08)	92.73 (68.84–140.25)	<0.001

BrSI, (median[IQR])	17.8 (8.98–30.86)	16 (8.41–29.27)	53.96 (30.55–73.88)	<0.001

BrSIG, (median[IQR])	245.67 (130.20–450)	237.5 (122.12–420.30)	647.63 (325–990.47)	<0.001

**Table 3:** Sensitivity, specificity, and optimal cut-point values in predicting mortality for shock indices in pediatric burn patients.

Predictor	Optimal Cut Point	Sensitivity (%)	Specificity (%)	PPV (%)	NPV (%)	AUC
SI	1.36	50.85	79.3	7.71	97.93	0.664
MSI	1.87	55.93	80.28	8.8	98.17	0.675
ASI	1.56	72.88	34.31	3.64	97.38	0.518
rSI	0.75	54.24	75.26	6.94	97.97	0.664
rSIG	9.90	59.32	90.02	16.83	98.49	0.770
BSI	21.79	83.05	79.64	12.19	99.28	0.872
BMSI	38.21	79.66	88.24	18.73	99.22	0.871
BASI	39.06	77.97	62.23	6.56	98.81	0.764
BrSI	15.13	83.05	73.07	9.5	99.22	0.851
BrSIG	159.54	86.44	57.09	6.42	99.2	0.782

**Abbreviations:** NPV, negative predictive value; PPV, positive predictive value; AUC, area under the curve

**Table 4:** Sensitivity, specificity, and optimal cut-point values in predicting mortality for shock indices in adult burn patients.

Predictor	Optimal Cut Point	Sensitivity (%)	Specificity (%)	PPV (%)	NPV (%)	AUC
SI	0.86	67.57	79.67	16.67	97.61	0.758
MSI	1.20	62.16	81.63	16.91	97.29	0.735
ASI	35.32	70.27	82.76	19.7	97.88	0.772
rSI	1.15	67.57	79.67	16.67	97.61	0.758
rSIG	17.5	72.97	74.63	14.75	97.87	0.780
BSI	18.46	91.89	78.7	20.61	99.38	0.888
BMSI	32.70	83.78	87.97	29.52	98.9	0.885
BASI	62.5	86.49	91.71	38.55	99.12	0.936
BrSI	30.5	75.68	77.24	16.67	98.14	0.826
BrSIG	325	78.38	63.58	11.46	97.99	0.755
